# Comparative Investigation of Cytotoxic Effects of Structurally Diverse Small Molecules and In Silico Analysis of 1‐Acetyl‐4‐(4‐Hydroxyphenyl)piperazine

**DOI:** 10.1111/jcmm.70890

**Published:** 2026-01-02

**Authors:** Azmi Yerlikaya, Emrah Tümer, Mustafa Güzel

**Affiliations:** ^1^ Department of Medical Biology, Faculty of Medicine Kutahya Health Sciences University Kutahya Turkey; ^2^ Department of Medical Genetics, Faculty of Medicine Kutahya Health Sciences University Kutahya Turkey; ^3^ Research Institute for Health Sciences and Technologies (SABITA), Center of Drug Discovery and Development İstanbul Medipol University Istanbul Turkiye; ^4^ Department of Basic Pharmaceutical Sciences School of Pharmacy, İstanbul Medipol University Istanbul Turkiye

**Keywords:** 1‐acetyl‐4‐(4‐hydroxyphenyl)piperazine, boronic acids, breast cancer, colon cancer, cytotoxicity

## Abstract

This study assessed the anticancer activity of boron‐containing and structurally diverse small molecules in 4T1 breast cancer and Caco‐2 colon adenocarcinoma cells. Initial screening showed that five boronic acids lacked significant cytotoxicity, underscoring the structural specificity required for boron‐mediated bioactivity. Similarly, reference compounds, including fumaric acid, caffeic acid, ferulic acid, dimethyl malonate and N‐(tert‐butoxycarbonyl)‐L‐alanine, showed no cytotoxic effect under identical conditions. Among the tested agents, 1‐acetyl‐4‐(4‐hydroxyphenyl)piperazine (1A4HP) displayed the most potent cytotoxicity, with IC_50_ values of 149.7 μM in 4T1 and 825 μM in Caco‐2 cells. For comparison, the clinically investigated antimetastatic agent tasquinimod showed moderate activity in 4T1 cells (IC_50_ = 180.7 μM), serving as a pharmacological benchmark. Mechanistic assays revealed that 1A4HP induced apoptosis and significantly impaired 4T1 cell migration, suggesting combined antiproliferative and antimetastatic effects. Computational analyses further supported 1A4HP's drug‐like potential by predicting favourable physicochemical properties, including balanced lipophilicity and high solubility. Molecular docking studies indicated a strong binding affinity to oestrogen receptor alpha (ERα), surpassing that of tamoxifen. Notably, despite 4T1's ER‐negative status, 1A4HP suppressed cell growth, suggesting possible ER‐independent or off‐target mechanisms, similar to tamoxifen's secondary effects. Collectively, these results identify 1A4HP as a promising lead compound for further exploration in breast cancers.

## 1 Introduction

Cancer is a group of diseases characterised by the uncontrolled proliferation of cells due to the disruption of normal regulatory mechanisms that govern cell behaviour. This uncontrolled growth leads to the invasion of healthy tissues and organs, and ultimately, metastasis throughout the body [1]. Globally, cancer ranks as the second leading cause of mortality, following cardiovascular diseases. It constitutes a significant public health burden, accounting for millions of deaths annually and affecting diverse populations across all age groups and geographic regions. Despite major advances in modern medicine and cancer biology, current therapeutic strategies are still far from being fully effective. Therefore, the development of novel compounds and innovative combinatorial treatment approaches remains an urgent priority. In 2012, approximately 8.2 million cancer‐related deaths were reported worldwide, with an estimated 14.1 million new cancer cases. This number is projected to rise significantly, reaching 22 million new cases annually over the next two decades [2]. Cancer is a multistep process resulting from genetic alterations that transform normal cells into highly malignant forms. Hanahan and Weinberg initially proposed six hallmarks of cancer, including self‐sufficiency in growth signals, insensitivity to growth‐inhibitory signals, evasion of apoptosis, limitless replicative potential, sustained angiogenesis and tissue invasion and metastasis [3]. With continued progress in the field, additional features have been incorporated into this framework, such as deregulated cellular metabolism (e.g., Warburg effect) and immune evasion [4]. Recent studies highlight the crucial role of epigenetic modifications, in addition to genetic changes, in regulating transcriptional activity and contributing to cancer pathogenesis. Key epigenetic mechanisms implicated in tumour initiation, progression and metastasis include aberrant DNA methylation, histone modifications and dysregulation of long non‐coding RNAs (lncRNAs) [5]. Histone acetylation, regulated by histone acetyltransferases (HATs) and histone deacetylases (HDACs), plays a central role in modulating gene expression [6, 7]. This study aimed to contribute to the development of novel, selective drug candidates targeting various cancer cells. The selected compounds—including fumaric acid, caffeic acid, 1‐(4‐hydroxy‐3‐methoxy)cinnamic acid, dimethyl malonate, N‐(tert‐butoxycarbonyl)‐L‐alanine, 1‐acetyl‐4‐(4‐hydroxyphenyl)piperazine, butylboronic acid, 4‐carboxyphenylboronic acid, 4‐aminocarbonylphenylboronic acid, 1‐butenylboronic acid and 3‐(2‐carboxyethyl)phenylboronic acid and tasquinimod—are investigated for their potential anticancer properties based on their distinct bioactive profiles. Fumaric acid and its phenolic analogs (caffeic acid and 1‐(4‐hydroxy‐3‐methoxy)cinnamic acid) exhibit pro‐apoptotic and anti‐inflammatory effects, with documented roles in ROS generation and NF‐κB pathway suppression, mechanisms known to impair cancer cell survival and proliferation [8, 9]. The boronic acid series—including butylboronic acid, 4‐carboxyphenylboronic acid, 4‐aminocarbonylphenylboronic acid, 1‐butenylboronic acid and 3‐(2‐carboxyethyl)phenylboronic acid—demonstrate pharmacological relevance through their electrophilic boron centers. Although these simpler boronic acids are structurally distinct from proteasome inhibitors like bortezomib, they may still interfere with cancer cell signalling by targeting serine hydrolases, binding to cell surface glycans or modulating enzyme families involved in tumour progression. This potential stems from the well‐established ability of boronic acids to form reversible covalent bonds with serine and threonine residues in proteases [10, 11]. As a malonate derivative, dimethyl malonate may disrupt cancer cell metabolism by competitively inhibiting succinate dehydrogenase or other key metabolic enzymes, resulting in energy stress. This metabolic disturbance is known to trigger ferroptosis, thereby suppressing the proliferation and migration of triple‐negative breast cancer (TNBC) cells [12, 13]. N‐(tert‐Butoxycarbonyl)‐L‐alanine, while primarily a synthetic intermediate, may serve as a precursor for peptide‐based therapeutics or boron‐conjugated analogs. The heterocyclic compound 1‐acetyl‐4‐(4‐hydroxyphenyl)piperazine (1A4HP) presents dual functionality—its aromatic moiety may intercalate with DNA while its piperazine core could modulate neurotransmitter‐linked receptors increasingly implicated in tumour growth regulation [14]. By systematically evaluating these structurally diverse compounds, we aim to identify novel agents that selectively target cancer cells through oxidative stress induction, metabolic interference, enzyme inhibition or receptor modulation, potentially yielding therapeutic alternatives with improved specificity and reduced toxicity to healthy cells compared to conventional chemotherapy. Surprisingly, among all compounds tested, 1A4HP demonstrated superior drug‐like properties and emerged as the most promising candidate for further investigation as a potential anticancer agent.

## 2 Material and Methods

### 2.1 Chemicals and Reagents

RPMI‐1640 and DMEM culture media, fetal bovine serum (FBS), trypsin, penicillin/streptomycin, dimethyl sulfoxide (DMSO), mitomycin C, serological pipettes, T25 cell culture flasks, 35 × 10 mm sterile petri dishes and Millipore Stericup Vacuum Filtration Systems were purchased from Sigma‐Aldrich (St. Louis, MO, USA). Cell Counting Kit‐8 (CCK‐8) was obtained from Roche Pharmaceuticals (Basel, Switzerland). Sodium butyrate (Cat. No: B5887), fumaric acid (Cat. No: 47910), caffeic acid (Cat. No: 205546), dimethyl malonate (Cat. No: 63380), 1‐(4‐hydroxy‐3‐methoxy)cinnamic acid (Cat. No: 537‐98‐4), 1‐acetyl‐4‐(4‐hydroxyphenyl)piperazine (Cat. No: 511943), N‐(tert‐butoxycarbonyl)‐L‐alanine (Cat. No: 15380), butylboronic acid (Cat. No: 163244), 4‐carboxylphenylboronic acid pinacol ester (Cat. No: 513490), 4‐aminocarbonylphenylboronic acid (Cat. No: 683876) and tasquinimod (Cat. No: SML2489) were obtained from Sigma‐Aldrich (St. Louis, MO, USA). 1‐Butenylboronic acid (Cat. No: sc‐208628) and 3‐(2‐carboxyethyl)phenylboronic acid (Cat. No: sc‐288640) were obtained from Santa Cruz Biotechnology Inc. (Heidelberg, Germany). 4T1 cells (ATCC CRL‐2539) and Caco‐2 cells (ATCC HTB‐37) were obtained from the American Type Culture Collection (ATCC, Manassas, VA, USA) and generously provided by Prof. Dr. Nuray Erin (Akdeniz University, Antalya, Turkey) and Prof. Dr. Miriş Dikmen (Anadolu University, Eskişehir, Turkey), respectively.

### 2.2 Cell Culture

RPMI‐1640 medium supplemented with 10% FBS was used for the culture of 4T1 breast cancer cells, a p53‐mutant and transplantable tumour cell line that is highly tumorigenic and invasive [15, 16]. Caco‐2 colon cancer cell lines were cultured in DMEM medium supplemented with 10% FBS; Caco‐2 cells are also p53‐deficient cell lines [17]. Wu et al. [18] reported that Caco‐2 monolayers cultured in DMEM are more reliable and stable than those in MEM, which is essentially the same as EMEM, Eagle's Minimum Essential Medium. DMEM supplemented with 10% FBS was therefore used for the cytotoxicity assay, ensuring more consistent growth and reliable assay outcomes [18, 19]. Cells were cultured in an HF90 incubator providing 37°C and a 5% CO_2_ environment. Stock cultures were maintained in 25 cm^2^ sterile Corning flasks, and experimental cultures were grown in 35 × 10 mm or 60 × 15 mm sterile petri dishes. Cell passage was typically performed at ~70% confluence. Cells were washed with 1 mL PBS; then, 2 mL of 0.25% trypsin (prepared in PBS) was added to the flasks, and cells were detached after approximately 3 min of trypsin treatment. After aspirating trypsin with a Pasteur pipette connected to a vacuum pump, cells were diluted 1:10 with fresh medium and seeded into flasks. Cell passage was performed periodically every 3 days [20].

### 2.3 Cell Viability Assay

To determine IC_50_ values, 10,000 cells were seeded in each well of 96‐well plates. Cells in logarithmic phase were treated with different concentrations of inhibitors/compounds (fumaric acid, caffeic acid, 1‐(4‐hydroxy‐3‐methoxy)cinnamic acid, dimethyl malonate, 1A4HP and N‐(tert‐butoxycarbonyl)‐L‐alanine, tasquinimod) as well as boron‐containing compounds (i.e., butylboronic acid, 4‐carboxyphenylboronic acid, 4‐aminocarbonylphenylboronic acid, 1‐butenylboronic acid, 3‐(2‐carboxyethyl)phenylboronic acid). Cells were treated for 24 h with concentrations ranging from 10 nM to 400 μM for most compounds and tasquinimod (10 nM—200 μM). After treatment, media were aspirated and 100 μL of DMEM or RPMI‐1640 containing 0.5% FBS and CCK‐8 was added to each well. After 2 h of incubation, absorbance was measured at 450 nm with 630 nm as reference using an ELISA reader. IC_50_ values were determined using GraphPad Prism 5.0 (*n* = 3) [21].

### 2.4 Cell Death Mode Analysis

We next investigated the effects of 1A4HP, bortezomib, isotonic solution (vehicle for bortezomib) or DMSO (vehicle for 1A4HP) on cell death mechanisms in 4T1 breast cancer cells following 48 h of treatment. Cell death was assessed using the acridine orange/ethidium bromide (AO/EB) staining method, a rapid and straightforward morphological assay for visualising and quantifying apoptosis and necrosis. The cells were seeded at a density of 20,000 cells per well in a 48‐well plate. After 24 h of seeding, the cells were treated with 75 μM 1A4HP, 36 μM bortezomib or inhibitor vehicles as indicated (*n* = 3). After 48 h of treatment, the culture medium was aspirated, and 100 μL of AO/EB staining solution (containing 100 μg/mL AO and 100 μg/mL EB) was added. The cells were incubated for 5 min, after which the staining solution was removed and replaced with 100 μL of PBS [22, 23]. Fluorescence images were immediately captured using a Zeiss Axio Vert A.1 microscope with a 20× objective (GFP filter, 475 nm). All images were saved as JPG files using ZEN 2.5 Pro software.

### 2.5 Wound Assay

4T1 murine breast cancer cells were grown in 35 × 10 mm sterile petri dishes. The cells were cultured to at least 90% confluence. Using a 200 μL sterile pipet tip, three separate wounds were scratched in each petri dish. Afterwards, the cells were treated with 0.5% DMSO (control), 75 μM 1A4HP and 36 μM bortezomib for 48 h. Mitomycin C (10 μg/mL) was added to all experimental plates to block cell proliferation [24]. The migration and morphology of cells were recorded with an inverted microscope using a 4× objective (AE21; Motic Europe, Barcelona, Spain) [25, 26, 27].

### 2.6 In Silico Analysis

The SMILES notation for 1A4HP (CC(=O)N1CCN(CC1)C2=CC=C(C=C2)O), computed using OEChem v2.3.0, was retrieved from the PubChem database. This structural representation was subsequently used for potential target prediction via the SwissTargetPrediction web tool (http://www.swisstargetprediction.ch/), which is designed to predict the most probable protein targets of bioactive small molecules in *Homo sapiens* and other vertebrate species [28]. The prediction was carried out using default parameters, with *Homo sapiens* selected as the target organism. The SwissADME web tool (http://www.swissadme.ch/index.php) was used to evaluate the pharmacokinetic and pharmacodynamic properties of 1A4HP. SwissADME enables the calculation of a wide range of physicochemical descriptors, and the prediction of ADME parameters, pharmacokinetic behaviours, drug likeness and medicinal chemistry friendliness of small molecules to support drug discovery efforts [29]. For molecular docking, the SMILES notation of 1A4HP was submitted as the ligand input to the SwissDock platform (https://www.swissdock.ch/), and docking was performed using the ‘Attracting Cavities’ protocol. Following ligand preparation, molecular docking of 1‐acetyl‐4‐(4‐hydroxyphenyl)piperazine (1A4HP) was performed against a panel of cancer and inflammation‐related protein targets, including oestrogen receptor alpha (ERα, PDB ID: 1ERE), histone deacetylase 2 (HDAC2, 4LY1), carbonic anhydrase IX (CA IX, 6FE2), carbonic anhydrase VII (hCA VII, 7NC4), cyclooxygenase‐2 (COX‐2, 6COX1), ribonucleotide reductase subunit M2 (hRRM2, 3OLJ), monoamine oxidase B (MAO‐B, 2V5Z), human farnesyltransferase (hFNTA, 1FT2), matrix metalloproteinase‐1 (MMP‐1, 1HFC), the human sigma‐1 receptor (SIGMAR1, 5HK1), and the TWIST1–TCF4–ALX4 complex (TTA, 8OSB). Where applicable, all protein chains and catalytically essential zinc ions (Zn^2+^) were retained during docking. Native ligands were removed prior to introducing 1A4HP. Docking grids were defined by centering on the active or catalytic sites of each target, for instance, at coordinates *X* = 34, *Y* = 47, *Z* = 90 Å with a grid size of 20 × 20 × 20 Å for docking to ERα. The cavity type was set to ‘buried’ to focus on internal binding pockets. Docking was carried out using one Random Initial Condition (RIC), and sampling exhaustivity was set to ‘medium’ to allow efficient yet focused exploration of relevant binding conformations [30, 31].

### 2.7 Statistical Analysis

Data were analysed using GraphPad Prism 5 software. Differences between groups were assessed by one‐way ANOVA followed by the Bonferroni's Multiple Comparison test. A *p*‐value of less than 0.05 was considered statistically significant. IC_50_ values were determined by nonlinear regression analysis as previously described [32].

## 3 Results

**FIGURE 1:**
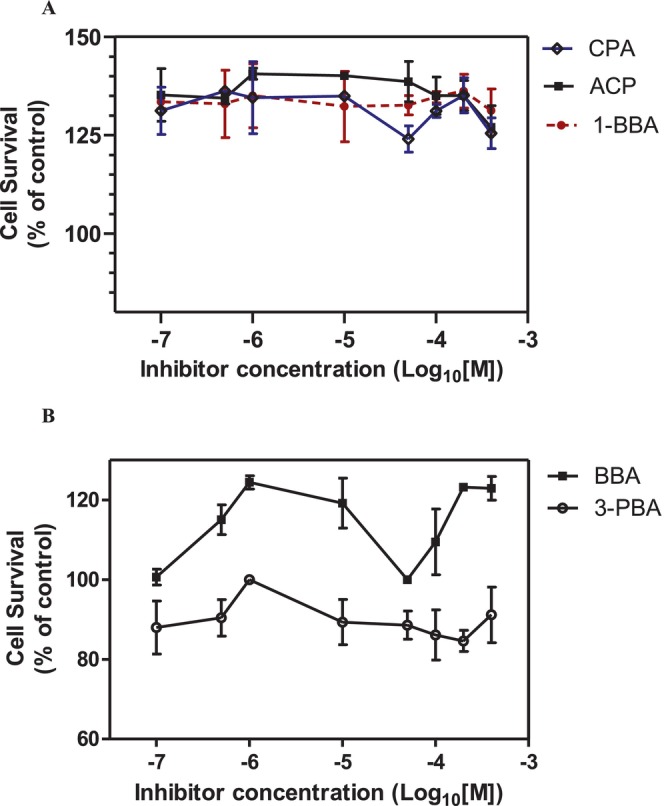
Cytotoxic effecs of boronic acid derivatives. (A) The effect of 4‐carboxyphenylboronic acid (CPA), 4‐aminocarbonylphenylboronic acid (ACP) and 1‐butenylboronic acid (1‐BBA). (B) The effect of butylboronic acid (BBA) and 3‐(2‐carboxyethyl)phenylboronic acid (3‐PBA). The cell viability was determined by CCK‐8 assay. Cells were treated for 24 h with concentrations ranging from 100 nM to 400 μM. The absorbance was measured at 450 nm with 630 nm as reference using an ELISA reader. IC_50_ values were determined using GraphPad Prism 5.0 (*n* = 3).

To explore the anticancer potential of boron‐containing compounds, we initially assessed a series of structurally diverse boronic acids, including butylboronic acid (BBA), 4‐carboxyphenylboronic acid (CPA), 4‐aminocarbonylphenylboronic acid (ACP), 1‐butenylboronic acid (1‐BBA) and 3‐(2‐carboxyethyl)phenylboronic acid (3‐PBA) (see Table 1 for chemical structures and properties). This investigation aimed to determine whether the incorporation of boron could enhance pharmacological activity across various cancer cell lines. We used a 24 h time point, widely regarded as a standard period for early cytotoxicity screening, to assess initial cytotoxic effects while minimising confounding factors associated with longer incubations, such as vehicle‐related effects (e.g., DMSO), nutrient depletion, secondary stress responses and changes in cell density. Surprisingly, none of the five tested boronic acid derivatives demonstrated measurable cytotoxicity against 4T1 breast cancer cells following a 24 h treatment at concentrations ranging from 100 nM to 400 μM (Figure 1A,B). While limited efficacy was anticipated given the rarity of clinically successful boron‐based agents, the complete lack of residual activity across all tested compounds was unexpected.

**TABLE 1:** Comparison of chemical structures of various molecules investigated for cytotoxic effects. The structures were drawn with BIOVIA Draw 2025.

Compound	Structure	Core structure	Functional groups	Key features
Fumaric acid	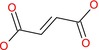	Unsaturated dicarboxylic acid	Two carboxyl groups (COOH), C=C double bond (trans)	Alkene with two COOH groups
Caffeic acid	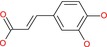	Hydroxycinnamic acid	Phenol (OH), carboxylic acid (COOH), alkene	3,4‐dihydroxyphenyl with propenoic acid side chain
4‐Hydroxy‐3‐methoxycinnamic acid	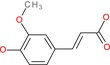	Hydroxycinnamic acid	Phenol (OH), methoxy (OCH3), carboxylic acid (COOH)	Similar to caffeic acid but methoxy at 3‐position
Dimethyl malonate	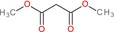	Malonic acid ester	Two ester groups (COOCH3)	Two methyl esters on malonate backbone
N‐(tert‐Butoxycarbonyl)‐L‐alanine	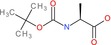	Amino acid derivative	Boc‐protected amine (carbamate), carboxylic acid	Boc protecting group on amino group
1‐Acetyl‐4‐(4‐hydroxyphenyl)piperazine	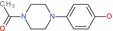	Piperazine derivative	Acetyl amide, phenol (OH)	Piperazine ring with acetyl and hydroxyphenyl substituents
Butylboronic acid	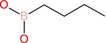	Alkyl boronic acid	Boronic acid (B(OH)2), alkyl chain (butyl)	Primary alkyl boronic acid
4‐Carboxyphenylboronic acid	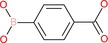	Aromatic boronic acid	Boronic acid (B(OH)2), carboxyl group (COOH)	Phenyl ring with para‐COOH and B(OH)2
4‐Aminocarbonylphenylboronic acid	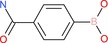	Aromatic boronic acid	Boronic acid (B(OH)2), amide (CONH2)	Phenyl ring with para‐carbamoyl and B(OH)2
1‐Butenylboronic acid	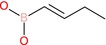	Alkene boronic acid	Boronic acid (B(OH)2), alkene (C=C)	Boronic acid attached to 1‐butenyl group
3‐(2‐Carboxyethly)phenylboronic acid	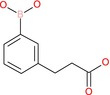	Aromatic boronic acid	Boronic acid (B(OH)2), carboxylic acid (COOH) on ethyl side chain	Phenyl ring with 3‐substituted 2‐carboxyethyl and boronic acid
Tasquinimod	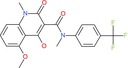	Quinoline derivative	Carboxylic acid, nitrogen heterocycle	Anti‐angiogenic agent with quinoline scaffold
Bortezomib	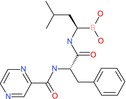	Peptide boronic acid	Boronic acid, amides, peptide bonds	Dipeptide boronic acid proteasome inhibitor

**FIGURE 2:**
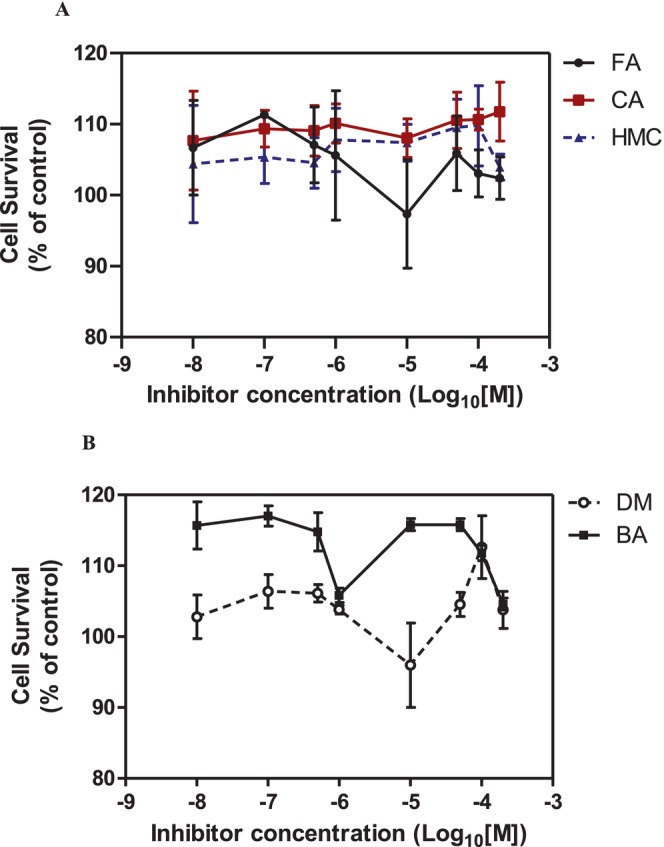
Cytotoxic effecs of various small molecules on 4T1 breast cancer cells. (A) Effect of fumaric acid (FA), caffeic acid (CA) and 1‐(4‐hydroxy‐3‐methoxy)cinnamic acid (HMC). (B) Effect of dimethyl malonate (DM) and N‐(tert‐butoxycarbonyl)‐L‐alanine (BA). The cell viability was determined by CCK‐8 assay. Cells were treated for 24 h with concentrations ranging from 10 nM to 400 μM for FA, CA, HMC, DM and BA. The absorbance was measured at 450 nm with 630 nm as reference using an ELISA reader. IC_50_ values were determined using GraphPad Prism 5.0 (*n* = 3).

We then evaluated the cytotoxic effects of fumaric acid, caffeic acid, 1‐(4‐hydroxy‐3‐methoxy)cinnamic acid (HMC, ferulic acid), dimethyl malonate and N‐(tert‐butoxycarbonyl)‐L‐alanine (Boc‐L‐alanine) (Table 1) in 4T1 breast cancer cells, a well‐established murine model of TNBC [16]. These compounds were selected for their structural diversity, incorporating features such as α, β‐unsaturated carbonyls, phenolic groups and ester functionalities—chemical motifs known to influence redox homeostasis, metabolic pathways and membrane permeability. By examining structurally related and distinct molecules, we aimed to explore how subtle structural variations affect cytotoxic responses, thereby supporting a structure–activity relationship (SAR)‐oriented approach. Following 24 h treatments across a concentration range of 10 nM to 400 μM, none of the tested compounds exhibited measurable cytotoxicity against 4T1 cells (Figure 2A,B, Table 1).

**FIGURE 3:**
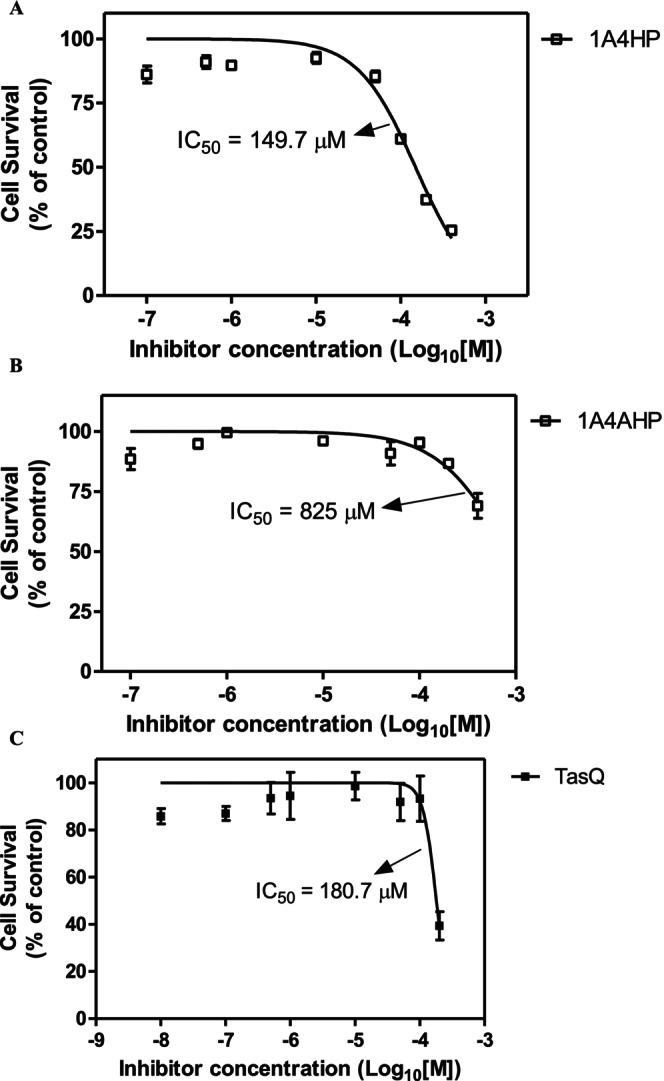
Cytotoxic effecs of 1A4HP on 4T1 breast cancer cells and Caco‐2 colon cancer cells. (A) Effect of 1A4HP in 4T1 cells, (B) Effect of 1A4HP in Caco‐2 cells. (C) The cytotoxic effect of tasquinimod (TasQ) in 4T1 cells. The cell viability was determined by CCK‐8 assay. Cells were treated for 24 h with concentrations ranging from 100 nM to 400 μM for 1A4HP and 10 nM—200 μM for TasQ. The absorbance was measured at 450 nm with 630 nm as reference using an ELISA reader. IC_50_ values were determined using GraphPad Prism 5.0 (*n* = 3).

We next assessed the cytotoxic activity of 1‐acetyl‐4‐(4‐hydroxyphenyl)piperazine (1A4HP) in 4T1 breast cancer and Caco‐2 colon cancer cell lines. The compound exhibited an IC_50_ of 149.7 μM in 4T1 cells and 825 μM in Caco‐2 cells (Figure 3A,B), indicating that 4T1 cells are more sensitive to 1A4HP. In the same experiment, we also evaluated the cytotoxicity of tasquinimod, which showed an IC_50_ of 180.7 μM in 4T1 cells (Figure 3C). Tasquinimod is a novel anti‐angiogenic and anti‐metastatic agent that modulates the tumour microenvironment and is currently undergoing Phase III clinical trials for the treatment of castration‐resistant prostate cancer [33]. Although its exact molecular target has not been clearly identified, Isaacs et al. [33] reported that tasquinimod acts as an allosteric modulator of HDAC4, a crucial regulator of epigenetic processes and survival pathways in the tumour microenvironment under stress.

**FIGURE 4:**
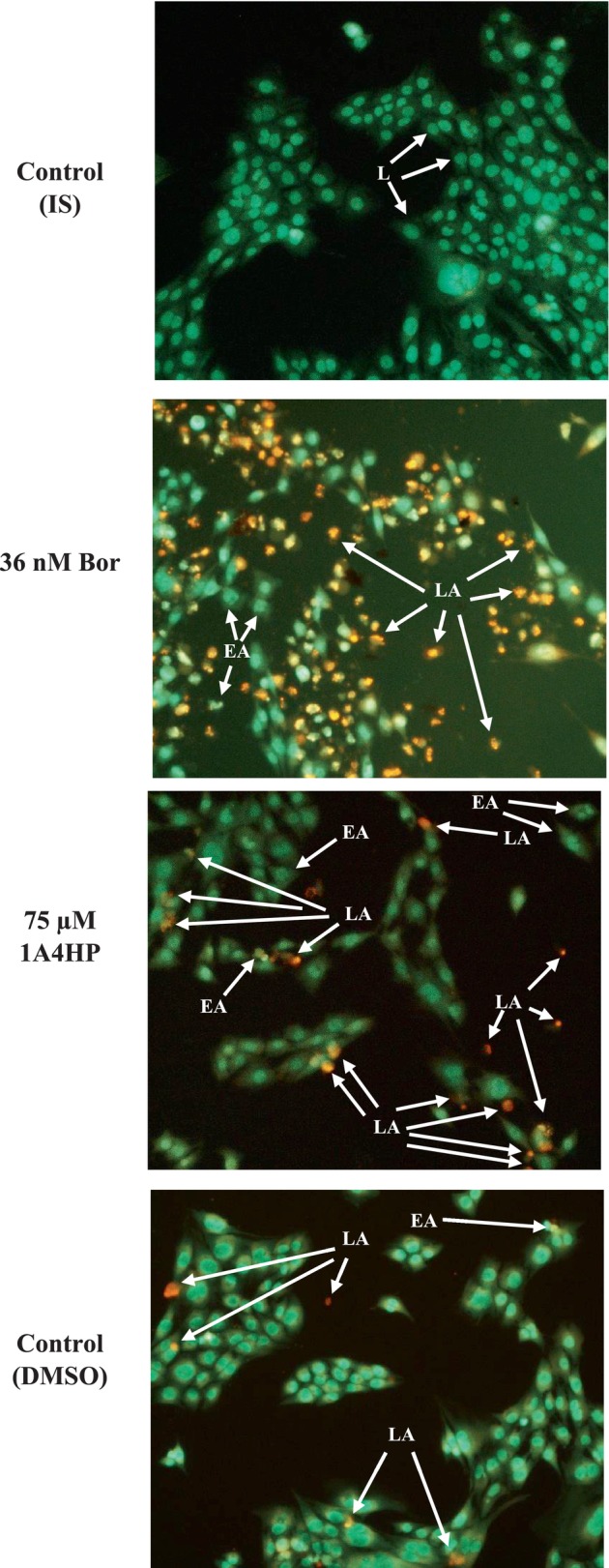
Cell death mode of 1A4HP in 4T1 cell. The cells were treated with 75 μM 1A4HP and 36 μM bortezomib (Bor) as a positive control for 48 h. After treatment, the apoptotic and necrotic cell populations were determined by dual AO/EB staining using a fluorescent Zeiss Axio Vert A.1 microscope with a 20× objective (GFP filter, 475 nm). All images were saved as JPG files using ZEN 2.5 Pro software.

Based on these encouraging results, we further examined the mode of cell death induced by 1A4HP in 4T1 cells using acridine orange/ethidium bromide (AO/EB) dual staining after 24 h of treatment. As shown in Figure 4, control cells treated with isotonic solution (vehicle for bortezomib as a positive control) or DMSO (vehicle for 1A4HP) showed minimal apoptotic or necrotic features. Most cells in these groups remained viable and exhibited uniform green fluorescence, indicating intact cell membranes. In contrast, treatment with 36 nM bortezomib—chosen based on our previously reported IC_50_ value of 71 nM in 4T1 cells [16]—resulted in a significant increase in early apoptotic (EA; bright green nuclear dots) and late apoptotic (LA; orange fluorescence with nuclear fragmentation due to EB incorporation) cells. Notably, treatment with 75 μM 1A4HP for 24 h also led to a marked increase in both EA and LA cell populations compared to the isotonic and DMSO controls (Figure 4). These results further support the pro‐apoptotic activity of 1A4HP and its potential as a promising anti‐cancer candidate.

**FIGURE 5:**
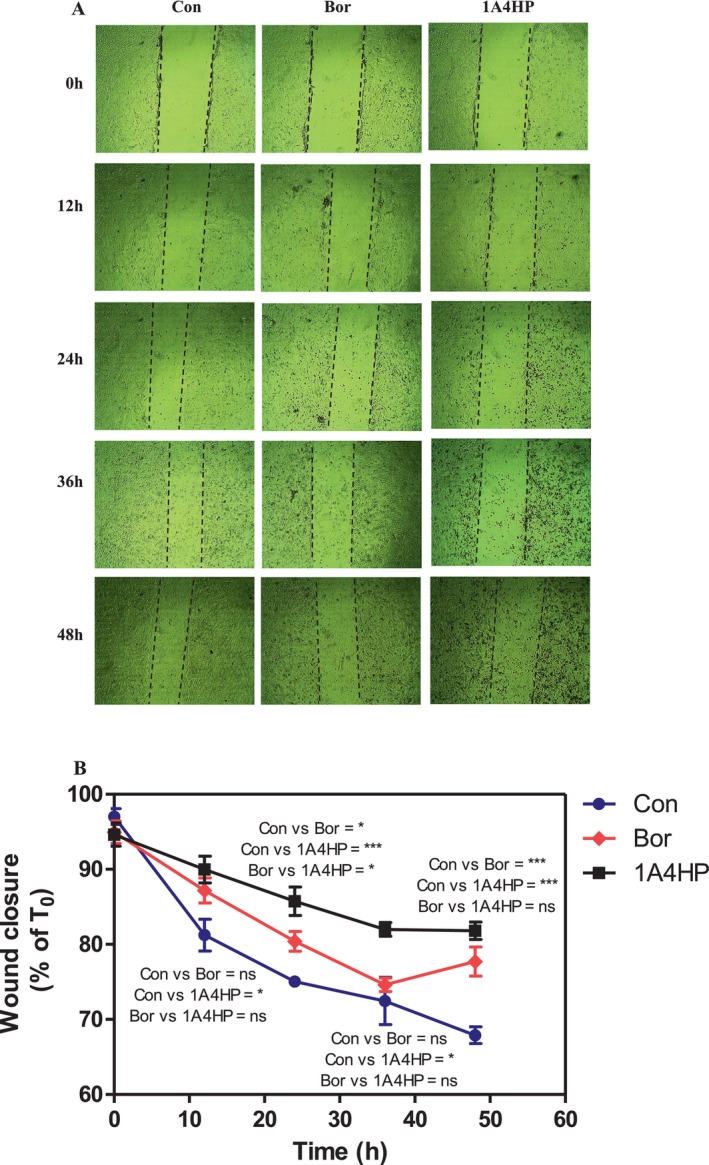
Evaluation of cell migration following 1A4HP treatment in 4T1 cells. (A) Representative images of wound closure following treatment with 1A4HP and bortezomib (Bor). Three independent scratch wounds were formed in each 35 × 10 mm sterile petri dish. The cells were then treated with 0.5% DMSO (control), 75 μM 1A4HP or 36 μM Bor for 48 h. To prevent cell proliferation, mitomycin C (10 μg/mL) was added to all treatment groups. Cell migration and morphological changes were observed at 12 h intervals using an inverted microscope equipped with a 4× objective (*n* = 3). (B) A graphical analysis of wound closure up to 48 h treatment period. Statistical analysis was performed using GraphPad Prism 5.0, applying One‐way ANOVA with Bonferroni's Multiple Comparison test. *p* < 0.05 is indicated by * and *p* < 0.001 by ***, ns, not significant.

We next assessed the effects of 1A4HP on the migratory behaviour of 4T1 breast cancer cells in comparison with bortezomib, using the in vitro scratch wound healing assay—a widely accepted method for evaluating cell migration capacity [34]. As shown in Figure 5A,B, cell migration was monitored for up to 48 h following scratch induction. To exclude the confounding influence of cell proliferation, cells were pretreated with mitomycin C (10 μg/mL) for 2 h prior to scratch formation. Afterward, they were maintained under serum‐free conditions in the presence of either 1A4HP (75 μM) or bortezomib (36 nM) throughout the 48 h observation period. Bortezomib was selected as a positive control due to its well‐established potency as a 26S proteasome inhibitor and its demonstrated cytotoxic effect on 4T1 breast cancer cells, with a low IC_50_ value of 71 nM, compared to conventional chemotherapeutic agents such as cisplatin and 5‐fluorouracil, which exhibited higher IC_50_ values of 14.2 and 8.9 μM, respectively [16, 32]. The experimental design ensured that any inhibition of wound closure was due to impaired cell migration rather than suppression of proliferation. Both 1A4HP and bortezomib significantly reduced cell migration across the 48 h period. For example, 1A4HP significantly inhibited wound closure at 12 h (*p* < 0.05), 24 h (*p* < 0.001), 36 h (*p* < 0.05) and 48 h (*p* < 0.001). The transient lack of statistical significance observed for bortezomib at the 36 h time point may be attributed to experimental variability or its known high cytotoxicity. Collectively, these findings indicate that 1A4HP, similar to bortezomib, markedly impairs the migratory potential of 4T1 breast cancer cells, suggesting a potential role in limiting metastatic progression.

**FIGURE 6:**
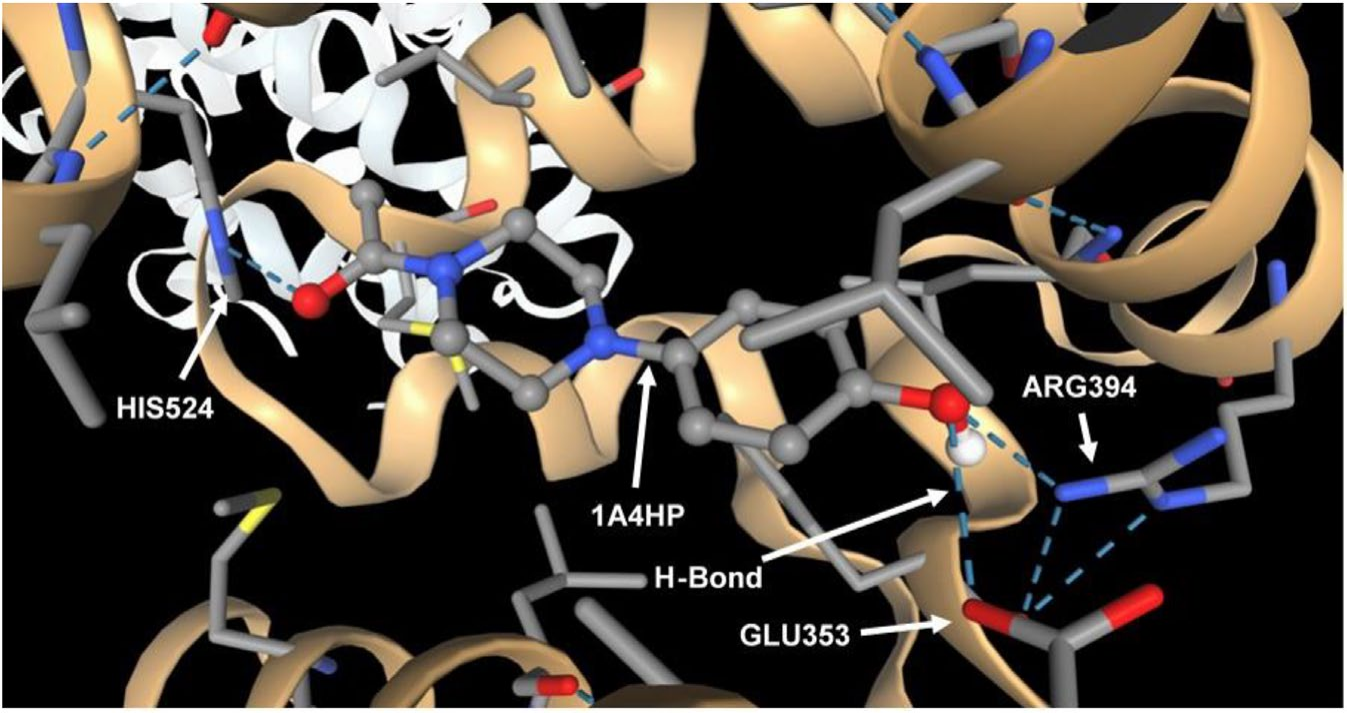
Molecular docking of 1A4HP to oestrogen receptor alpha (ERα). The SMILES notation of 1‐acetyl‐4‐(4‐hydroxyphenyl)piperazine (1A4HP) (CC(=O)N1CCN(CC1)C2=CC=C(C=C2)O) was obtained from the PubChem database and generated using OEChem v2.3.0. The three‐dimensional structure of ERα (PDB ID: 1ERE) was retrieved from the RCSB Protein Data Bank (RCSB.org). Molecular docking was conducted using the ‘Attracting Cavities’ protocol available on the SwissDock platform to predict the binding interaction between 1A4HP and the ERα active site.

Using in silico analyses, we then assessed the drug‐likeness and predicted molecular targets of 1A4HP to elucidate its potential mechanisms underlying cytotoxicity and inhibition of cell migration. As presented in Table 2, the compound 1A4HP exhibits favourable physicochemical and pharmacokinetic characteristics, indicating its potential as a viable drug candidate. These properties were evaluated using SwissADME, a platform designed to predict pharmacokinetics, drug‐likeness and medicinal chemistry friendliness of small molecules [29]. With a molecular weight of 220.27 g/mol and a consensus Log *P*_*o/w*_ of 0.98, 1A4HP demonstrates moderate lipophilicity and high aqueous solubility (Log *S* = −1.96). It complies fully with Lipinski's Rule of Five, possesses a bioavailability score of 0.55, and shows high predicted gastrointestinal absorption. Furthermore, it is not identified as a P‐glycoprotein (P‐gp) substrate or an inhibitor of CYP1A2, and it does not trigger Pan Assay Interference Compounds (PAINS) alerts. Its low synthetic accessibility score (1.51) suggests that it can be synthesised with relative ease. Although its molecular weight is slightly below the threshold for lead likeness, the compound's overall in silico profile warrants further exploration in the context of drug development. To identify the potential targets of 1A4HP, we utilised SwissTargetPrediction, a tool designed for the efficient prediction of protein targets for small molecules [28]. The SwissTargetPrediction analysis revealed that the potential targets of 1A4HP are primarily lyases and Family A G protein‐coupled receptors, each accounting for 33.3% of the predicted targets, followed by enzymes (20%), and membrane receptors and kinases, each representing 6.7%. Based on the findings, the proteins and enzymes listed in Table 3 were selected for molecular docking studies. Docking simulations were performed using SwissDock, a platform that integrates the Attracting Cavities 2.0 (AC) and AutoDock Vina algorithms [30, 35] to predict potential binding interactions between 1A4HP and its target proteins. Initial binding affinity and interaction strength were assessed using the Attracting Cavities 2.0 methodology. For each docking simulation, binding efficacy was evaluated by comparison with the corresponding native ligand, which served as a positive control. Notably, the analysis demonstrated that 1A4HP exhibited a higher binding affinity for oestrogen receptor alpha (ERα) than tamoxifen, a well‐characterised ligand for oestrogen receptors (ERα and ERβ) that functions as a selective oestrogen receptor modulator (SERM), displaying agonist activity in tissues such as the uterus and bone while acting as an antagonist in breast tissue [36]. The SwissParam docking score, which estimates the binding free energy (ΔG) of a ligand to its target, was −7.7229 kcal/mol for 1A4HP compared to −6.4979 kcal/mol for tamoxifen (Table 3, Figure 6), suggesting a stronger predicted interaction between 1A4HP and ERα. To validate these results, we further employed the AutoDock Vina algorithm within SwissDock, which corroborated the finding that 1A4HP binds more tightly to ERα than tamoxifen, with predicted binding affinities of −7.523 and −3.459 kcal/mol, respectively. In contrast, when the same computational approach was applied to evaluate 1A4HP binding to a range of other potential molecular targets, including histone deacetylase 2 (HDAC2, 4LY1), carbonic anhydrase IX (CA IX, 6FE2), carbonic anhydrase VII (hCA VII, 7NC4), cyclooxygenase‐2 (COX‐2, 6COX1), ribonucleotide reductase M2 subunit (hRRM2, 3OLJ), monoamine oxidase B (MAO‐B, 2V5Z), human farnesyltransferase (hFNTA, 1FT2), matrix metalloproteinase‐1 (MMP‐1, 1HFC), human sigma‐1 receptor (SIGMAR1) and TWIST1‐TCF4‐ALX4 complex (TTA, 8OSB), no significant binding interactions were observed relative to the pharmacological ligands of these targets (Table 3). These findings underscore the specificity and robustness of the predicted interaction between 1A4HP and ERα, warranting further investigation into its potential pharmacological implications.

**TABLE 2:** Physicochemical and drug‐likeness properties of 1A4HP.

Physicochemical properties	Formula	C_12_H_16_N_2_O_2_
	Molecular weight	220.27 g/mol
	Fraction Csp3	0.42
	Num. H‐bond acceptors	2
	Num. H‐bond donors	1
Lipophilicity	Log *P*_o/w_ (iLOGP)	1.90
	Consensus Log *P*_o/w_	0.98
Water solubility	Log *S* (ESOL)	−1.96
	Class	Very soluble
Pharmacokinetics	GI absorption	High
	BBB permeant	No
	Log *K*_p_ (skin permeation)	−6.96 cm/s
	P‐gp substrate	No
	CYP1A2 inhibitor	No
Druglikeness	Lipinski	Yes; 0 violation
	Bioavailability Score	0.55
Medicinal chemistry	PAINS	0 alert
	Synthetic accessibility	1.51
	Leadlikeness	No; 1 violation: MW < 250

*Note:* The in silico analysis was performed using SwissADME, with the SMILES notation of 1A4HP retrieved from PubChem. Fraction Csp3, the fraction of carbons in the sp3 hybridisation. Log *S* is the base‐10 logarithm of a molecule's solubility in water (mol/L).

Abbreviations: BBB, blood‐brain barrier; PAINS, Pan‐assay interference compounds.

**TABLE 3:** Molecular docking of 1A4HP with various proteins and enzymes.

Target	PDB ID	Ligand	AC Score	SwissParam Score
Oestrogen receptor alpha (ERα)	1ERE	1A4HP	−11.891142	−7.7229
		Tamoxifen	62.022516	−6.4979
Histone deacetylase 2 (HDAC2)	4LY1	1A4HP	1.057976	−6.8643
		ZN5	−1.192637	−7.8956
Carbonic anhydrase IX (CA IX)	6FE2	1A4HP	0.679736	−7.0074
		SLC‐0111	−105.304629	−7.3672
Carbonic anhydrase VII (hCA VII)	7NC4	1A4HP	10.989952	−5.9593
		U7Z	−10.362134	−6.8765
Cyclooxygenase‐2 (COX‐2)	6COX1	1A4HP	−1.789797	−6.9526
		SC‐558	−9.545584	−9.3061
Ribonucleotide reductase subunit M2 (hRRM2)	3OLJ	1A4HP	8.500998	−5.9788
		Osalmid	−12.513717	−6.0029
Monoamine oxidase B (MAO‐B)	2V5Z	1A4HP	−1.848713	−7.2709
		Safinamide	10.320823	−8.6026
Human farnesyltransferase	1FT2	1A4HP	4.076244	−6.6235
		Tipifarnib	3.841373	−8.4606
Matrix metalloproteinase‐1 (MMP‐1)	1HFC	1A4HP	10.785293	−5.9187
		CGS‐27023	18.248010	−7.0259
Human sigma‐1 receptor bound	5HK1	1A4HP	5.967787	−6.3808
		PD144418	5.486164	−7.3608
TWIST1‐TCF4‐ALX4 komplex	8OSB	1A4HP	5.033712	−6.4499
		LF3	−57.351417	−7.6641

*Note:* The protein structures (PDB IDs) were sourced from RCSB Protein Data Bank (RCSB PDB). The ligand, 1A4HP, was prepared for docking using its SMILES notation. For each target, the PDB structure was processed by removing any co‐crystallised ligands before running the docking algorithm. Essential heteroatoms (e.g., Zn) were retained in the target structures where applicable. AC Score is a scoring function that accounts for all energy contributions, making it ideal for ranking different poses of the same ligand bound to a single target (docking score). SwissParam Score provides an estimated binding free energy and is more appropriate for comparing different ligand/target combinations, rather than ranking multiple conformations of a single ligand.

## 4 Discussion

There remains an unmet need for effective cancer treatments, largely due to the global rise in cancer incidence and the frequent development of resistance mechanisms following prolonged use of conventional chemotherapeutic agents. In this study, we initially evaluated the cytotoxicity of five structurally distinct boronic acids. Our rationale was based on the clinically validated mechanism of the FDA‐approved boron‐containing drug bortezomib, which exerts its anticancer effect through reversible covalent binding of its boronic acid moiety to the catalytic threonine residue of PSMB5, the chymotrypsin‐like subunit of the 26S proteasome [37, 38]. Despite ongoing interest in boron‐based drug development, bortezomib remains the only approved boron‐containing anticancer agent, primarily used for the treatment of multiple myeloma and mantle cell lymphoma [39, 40, 41]. The cytotoxic effects of boron‐containing compounds were initially tested in the 4T1 murine mammary carcinoma cell line. As a triple‐negative, p53‐mutant model with high metastatic potential, 4T1 provides a more clinically relevant platform for studying p53‐independent apoptosis than p53 wild‐type models such as MCF‐7. This phenotype closely parallels that of the highly aggressive human MDA‐MB‐231 line. Moreover, in syngeneic BALB/c mice, 4T1 tumours grow rapidly and metastasize spontaneously, closely mimicking stage IV human breast cancer. Together, these characteristics make the 4T1 model a powerful and versatile tool for translational research, enabling seamless integration of in vitro mechanistic studies with in vivo validation. None of the compounds exhibited significant cytotoxic effects after 24 h of exposure. These findings highlight the importance of precise structural and mechanistic features for boron‐based anticancer activity, suggesting that effective bioactivity cannot be achieved through simple boronic acid substitution alone. This limited efficacy observed aligns with the current clinical landscape, where bortezomib (Velcade) remains one of the few FDA‐approved boron‐containing anticancer agents. Approved in 2003, bortezomib is a first‐in‐class boron‐containing proteasome inhibitor that selectively targets the chymotrypsin‐like activity of the β5 subunit (PSMB5) within the 20S proteasome core. It has demonstrated significant clinical efficacy in the treatment of multiple myeloma (MM) and mantle cell lymphoma [41, 42]. This success has spurred further exploration of boron in drug development, exemplified by ixazomib (Ninlaro), an orally bioavailable boronate ester approved in 2015 for relapsed/refractory MM. Like bortezomib, ixazomib preferentially inhibits the 20S proteasome's chymotrypsin‐like activity but offers improved pharmacokinetics [43]. Parallel screening of non‐boron compounds—fumaric acid, caffeic acid, HMC (ferulic acid), dimethyl malonate and N‐(tert‐butoxycarbonyl)‐L‐alanine (Boc‐L‐alanine)—similarly revealed no measurable cytotoxicity in 4T1 cells under identical conditions. While previous studies have reported limited or no cytotoxic effects for fumaric acid, dimethyl malonate and N‐(tert‐butoxycarbonyl)‐L‐alanine, both caffeic acid and HMC (ferulic acid) have been described as cytotoxic in other cancer cell models [44, 45, 46, 47]. The findings presented here thus suggest that the cytotoxic effects of caffeic acid and HMC may be cell line‐specific or influenced by treatment duration and/or concentration, underscoring the need for further investigation. For example, a 2021 study demonstrated that ferulic acid's cytotoxicity in MDA‐MB‐231 cells required 48 h exposure and suggested that it may be a vital anticancer agent, particularly for breast cancer, through its induction of apoptosis in a p53‐dependent manner [44]. The IC_50_ value of caffeic acid in the HCT116 cells was calculated as 826.59 μM at 24 h; however, when the effect of caffeic acid on Caco‐2 and HT‐29 cells was examined, it was observed that cell viability did not decrease below 50% in the applied dose range after 24 h, highlighting cell line variability [47]. In contrast to these results, it was determined that 1‐acetyl‐4‐(4‐hydroxyphenyl)piperazine (1A4HP) exhibited potent cytotoxicity, with IC_50_ values of 149.7 μM in 4T1 cells. On the other hand, the IC_50_ of 1A4HP on Caco‐2 cells was estimated at approximately 825 μM, considerably higher than typical chemotherapeutic agents, which may reflect the slower proliferation rate of Caco‐2 cells compared to 4T1 cells (doubling times: 42.5 h vs. 22.9 h, respectively) [16, 48]. In agreement, Bazzocco et al. [49] reported a doubling time of 51.4 h for Caco‐2 cells during the exponential growth phase. In parallel experiments, tasquinimod—an investigational anti‐angiogenic agent evaluated in Phase III trials for prostate cancer—exhibited moderate cytotoxic activity in 4T1 cells (IC_50_ = 180.7 μM). This aligns with prior reports indicating that tasquinimod's cell viability effects typically occur at micromolar concentrations, consistent with its primary mechanism of action targeting tumour microenvironment modulation (e.g., angiogenesis, immune evasion) via HDAC4 inhibition rather than direct tumour cell killing [33]. Therefore, we decided to investigate the cytotoxic mechanism of 1A4HP using acridine orange/ethidium bromide (AO/EB) dual staining, which confirmed that 1A4HP induced apoptosis, with significant increases in early and late apoptotic populations compared to vehicle controls. Furthermore, scratch wound assays demonstrated that 1A4HP (75 μM) significantly impaired 4T1 cell migration over 48 h, suggesting anti‐metastatic potential independent of proliferation. In silico analysis using SwissADME revealed that 1A4HP exhibits promising drug‐like properties, including moderate lipophilicity (Log *P* = 0.98), ensuring a balance between membrane permeability and solubility and high aqueous solubility (Log *S* = −1.96), supporting favourable dissolution and absorption. These characteristics highlight its potential as a viable drug candidate for further development. In agreement with these findings, molecular docking (SwissDock) predicted strong binding affinity for oestrogen receptor alpha (ERα), with a calculated ΔG of −7.72 kcal/mol for 1A4HP versus −6.50 kcal/mol for tamoxifen. AutoDock Vina validation corroborated these results (−7.52 kcal/mol vs. −3.46 kcal/mol), suggesting potential ERα modulation. Oestrogen receptor alpha (ERα) is a critical nuclear transcription factor that drives hormone‐dependent cancer progression—particularly in breast, ovarian, colorectal, prostate and endometrial cancers—by promoting tumour cell proliferation, survival and metastasis through genomic transcription and rapid kinase signalling pathways. Due to its central role, ERα is a key therapeutic target in oncology [50]. Interestingly, the 4T1 cell line, a TNBC model, lacks expression of oestrogen receptor (ER), progesterone receptor (PgR) and human epidermal growth factor receptor 2 (HER2) [51]. Despite this ER‐negative status, we observed significant growth inhibition in these cells. Further literature review revealed that tamoxifen, a well‐known ERα ligand, also suppresses ER‐negative breast cancer cell invasion and metastasis by accelerating Twist1 degradation [52]. Our findings align with this observation, indicating that 1A4HP may exhibit efficacy in both ER‐positive and ER‐negative cell lines. The results further suggest that both tamoxifen and 1A4HP may have off‐target mechanisms—beyond ER modulation—that contribute to cell growth inhibition and suppression of migration.

## Author Contributions

**Azmi Yerlikaya:** conceptualisation (lead), data curation, formal analysis, funding acquisition, investigation, writing – original draft, writing – review and editing. **Emrah Tümer:** conceptualisation, data curation, formal analysis, funding acquisition, investigation, writing – original draft, writing – review and editing. **Mustafa Güzel:** conceptualisation, data curation, formal analysis, investigation, writing – original draft, writing – review and editing.

## Ethics Statement

The authors have nothing to report.

## Conflicts of Interest

The authors declare no conflicts of interest.

## Data Availability

The data that support the findings of this study are available from the corresponding author upon reasonable request.
